# Electronic Health Record Portals and Patient-Centered Outcomes in CKD

**DOI:** 10.1016/j.xkme.2021.02.003

**Published:** 2021-03-06

**Authors:** Soraya Arzhan, Christos Argyropoulos, Maria-Eleni Roumelioti

**Affiliations:** Renal and Electrolyte Division, University of New Mexico, Albuquerque, NM

Related article, p. 231

With ongoing health care reform efforts in the United States, focus is shifting to the quality of health care delivery.^1^ Standardized quality indicators have been developed and proposed to measure key components of care across the full continuum of care delivery, including patient-centered outcomes, defined as outcomes meaningful and important to patients and caregivers (patient-centered outcomes).^2^ In recent years, electronic health record (EHR) measures of patient-centered outcomes and comparative effectiveness research have been integrated.^3^ The relationships between patient portal use and outcomes have been evaluated in different clinical settings and disease processes.^4^, ^5^, ^6^, ^7^

EHR and patient-centered outcome measures are important tools for encouraging patient engagement in health care, informing clinical decision making, and improving patient care (Box 1). Patient-provider communication is essential for favorable outcomes, and patients increasingly are encouraged to communicate with their health care providers through internet-based portals tethered to the EHR.^5^ Patient-centered outcome measures are one means of systematically gathering meaningful subjective information for patient care, population health, and research.^8^ It has been shown that patients who are more engaged in decision making and collaboration with health care providers can have better outcomes.^9^ Especially in chronic diseases, it is important that patients have more access to their own medical records in a simple way. Streamlining patient access to medical records plays an important role in patients feeling that they have more control over the treatment or management of their own chronic conditions.

Box 1 **Benefits and Pitfalls of EHR Use**

***Patient Care***

Increased adherence to guideline-based care
Enhanced surveillance and monitoring
Decreased medication errors
Improved quality of patient care by physicians
Improvement in vaccination rates
Facilitates smoking cessation
Benefits in primary care settings (eg, improved cholesterol management in diabetic patients)
Public health alerts: useful in reporting and recommending specific tests as well as suggesting secondary prevention

***Research***

Generation of samples for case-control studies
Construction of a cohort
Identification of people with certain conditions or outcomes
Large-scale high-throughput genetic research
Improved aggregation of patient data for quality improvement or clinical research
More reliable longitudinal clinical data
Examination of costs incurred for various CKD management aspects

***CKD Care***

Early identification/diagnosis of CKD and high-risk patients
Improved quality of care
Improved referral rates to nephrology clinics
Efficient exchange of information between caregivers and improved multidisciplinary collaboration
Timely referrals for dialysis access and renal transplantation
CKD stage-specific goals of care and CKD patient education
Medication monitoring and dose adjustment
National CKD surveillance to improve health through broader availability of population-level CKD data

***Patient Benefits***

Access, management, and sharing of personal health information with health care providers through secure messaging
Request of prescription renewals, viewing health summaries, reviewing test results, and accessing current list of medications
Receiving sex- and age-based automated important health reminders

***Pitfalls***

Increased work tasks; computerized order entry
Fragmentation of data
Loss of communication
Rigid clinical decision support, outdated content, alert fatigue
Human and capital resources required rendering inequalities among health care settings
Privacy and protection of personal health information

Abbreviations: CKD, chronic kidney disease; EHR, electronic health record.Data from references.^14^^,^^15^

Although chronic kidney disease (CKD) is common and associated with considerable morbidity and mortality, particularly among disadvantaged patients,^10^ many patients remain unaware of their diagnosis. EHR patient portals are one way to facilitate patient-provider communication and information sharing and help patients be more engaged in their CKD care through regular follow-up appointments, frequent tests, dissemination of test results, and receipt of education about CKD and CKD management, including lifestyle modifications.^11^, ^12^, ^13^ Providing portal access to disadvantaged patients with CKD affords them opportunities to become aware of their CKD and its progression. Furthermore, such access may encourage patients to be in closer contact with their health care providers while facilitating adherence to treatment and management modalities for comorbid conditions, CKD, and its complications.^14^^,^^15^

EHR and technology use can improve patient-centered outcomes in patients with CKD; notably, the association between the use of EHR portals by nondialysis patients with CKD and patient-centered outcomes is an important area that needs more research.^6^^,^^7^^,^^11^^,^^13^ In this issue of *Kidney Medicine*, Tome et al^7^ conducted a cross-sectional survey (between April 2015 and March 2018) of 245 nondialysis patients with CKD from nephrology clinics within 1 large academic medical center. They explored whether patient use of an EHR portal was associated with demographics or kidney function and whether portal use predicted patient-centered outcomes such as CKD-specific knowledge, CKD-related stress, and two patient self-ratings of health status.

An important finding of this study was that 65% of patients reported using the EHR portal to check their laboratory test results, manage appointments, message their providers, view their medical history, review educational resources, and renew prescriptions. They found that African Americans (odds ratio [OR], 0.34; 95% CI, 0.16-0.72 vs White patients), those with less formal education (OR, 0.06; 95% CI, 0.01-0.36), and those with lower income (OR, 0.28; 95% CI, 0.13-0.60; and OR, 0.26; 95% CI, 0.12-0.54 comparing income <$25,000 and $25,000-$50,000, respectively, with ≥$50,000) had lower odds of EHR portal use in univariate analysis. After adjustment for these factors, only income was associated with lower EHR portal use. Another important finding in univariable analysis was that portal users had higher CKD-specific knowledge (*P* = 0.02), higher ratings of current health (*P* = 0.03), and a trend for lower CKD-related stress (*P* = 0.05). Interestingly, kidney function (estimated glomerular filtration rate) was not associated with EHR portal use.

The findings of this study are an extension of previous work by Harrison et al^16^ conducted in Calgary, Alberta, Canada, between 2013 and 2014 at a multidisciplinary CKD clinic. In this study, a self-administered paper-based patient survey was used on 63 non–dialysis-dependent patients with CKD to determine perceptions of EHRs and identify factors that were associated with intention to use the EHR portal. In that report, most patients expressed their intention to use their portal (70%). Older patients with CKD (aged >65 years) were less likely to intend to use an EHR compared with younger patients with CKD (OR, 0.22; 95% CI, 0.06-0.78), whereas those with postsecondary education (OR, 3.31; 95% CI, 1.06-10.41) and internet access (OR, 5.70; 95% CI, 1.64-19.81) were more likely to intend to use an EHR portal. Greater involvement in their own care (50%), better access to laboratory results (76%), and access to health information (57%) were among the perceived benefits. In both studies, older patients and those with less formal education were less likely to use their portal. Although both studies were limited by their small sample size, the study by Tome et al has the advantage of measuring concurrently patient-centered outcomes, income, and kidney function.

In another interesting study originating from the United Kingdom, Phelps et al^17^ longitudinally investigated the Renal Patient View (RPV) use by patients over 4 years, as well as factors associated with more persistent RPV use. RPV is a system that allows patients with CKD access to test results and information about their health status and personal treatment. An impressive quantitative evaluation of 14,000 patient-years of access data took place in this study. The investigators found that a large proportion of patients with CKD regularly used their online EHRs. Patients were strongly interested in recent test results and clinic letters, while access to timely blood test results was a key factor driving the use of the RPV. Initial patient support increased persistent patient use.^17^

Agarwal et al^18^ explored how individual and environmental factors influenced the intentions to use the EHR and showed that patients who were more satisfied with their provider and felt more empowered and more actively involved had higher EHR use intentions. Their findings highlight the importance of communication tactics and technology characteristics in influencing patients’ intentions to access and use their personal EHRs.^18^ Both studies explore different aspects and factors of EHR portal use and complement the findings in the study by Tome et al.

Older age is associated with lower EHR use in patients with CKD,^6^^,^^13^^,^^16^^,^^17^ an association not seen in the study by Tome et al. With a study population having a mean age of 60 ± 17 years, the only age-related effect mentioned was that younger age (*P* = 0.02) was significantly associated with more patient-perceived CKD stress. The cross-sectional design of the study and limited sample size could be a possible explanation. In general, patients with CKD are usually older and more selective in their uptake of modern information technologies, while their level of education and knowledge of EHR functionality is lower compared with younger patients. In this regard, previous studies found that older patients who identified the benefits of greater personal engagement in their health care and of higher access to health information and laboratory results had a higher tendency to use EHR portals.^16^^,^^18^

Older adults are still underusing patient portals to engage with their health care providers. Engaging older adults requires more than just signing them up for patient portals. Creativity, simplicity, and confidentiality are crucial factors for successful engagement. Making patient portals more user friendly for older adults is essential. Getting people signed up is the first step, with the next crucial step being to walk them through the features of a portal. Patient portal access needs to make sense. It is important to explain the types of communications that would be easier through the patient portal while still encouraging telephone communications for emergencies and medical questions. Portals should be viewed as an added benefit and not as a replacement of the in-person relationship. Health care organizations can also take advantage of times when older patients are in the office or in the waiting room to explain the benefits of patient portals to them in person.

In addition to older age, various other barriers may limit the extent of EHR portal use. Patients with a chronic disease and a lower income may not be able to afford digital electronic devices and may not have consistent internet access. Patients with less formal education and those with fewer socioeconomic resources are particularly vulnerable for health care disparities, and the benefits of EHR portal use in this population remain unclear.^5^^,^^7^

Prior studies have shown that African Americans, older patients, and patients whose primary insurance is Medicaid are less likely to use portals compared with White patients, younger patients, and those with other forms of insurance.^6^^,^^13^ These findings were mirrored in the investigation by Tome et al. These results imply that in order to reduce disparities in care regarding health portals, some interventions targeting older individuals, African Americans, and patients with lower incomes are needed.^7^ Finally, limited patient education regarding how to apply for EHR portal access and use of up-to-date technologies (eg, web-based education, short message service texting, CKD-tailored mobile applications, Telehealth/Tele-nephrology, and interactive voice response system–based applications^11^), potential differences in attitude and perception regarding patient portal use, and confidentiality issues are some of the hurdles.^5^^,^^7^^,^^19^

Health portals should be accessible to all because patient-provider communication is a crucial aspect of care for disadvantaged patients who prefer this method of communication. A main issue is that although many health systems develop patient portals, there is a substantial portion of patients who are not receiving the expected gains from them, with some of the major reasons being differences in internet access, computer literacy, and internet proficiency, as well as patient attitudes. Hence, although well intended, portals may widen disparities in care for those most vulnerable instead of improving access to care for all.^7^ Moreover, there may be even more pronounced access problems in patients living in rural communities or those with limited financial means who often lack home computers or internet services. These factors can lead to limited or less access to consistent widely available internet for the portals that largely depend on internet access.^5^^,^^20^ Future research should explore how EHRs can be used to improve CKD care and research for individual patients, health systems, and populations.
